# Supplementary material for: Stability Characterization of the Novel Anti-Cancer HM-10/10 HDL-Mimetic Peptide
Source: Int J Mol Sci. 2023 Jun 13;24(12):10054. doi: 10.3390/ijms241210054 (PMC10298982; doi:10.3390/ijms241210054)
Supplement: Supplementary file 1 [file ijms-24-10054-s001.zip › ijms-2374640-supplementary.pdf]

SUPPLEMENTARY MATERIAL

Determination of Stability of HM1010 in CD-1 Mouse, Sprague-Dawley Rat, Beagle Dog, Cynomolgus Monkey, and Human Liver Microsomes and Cryopreserved Hepatocytes

Report No. ZXAA-0007-DV-HB-RPT-01
Testing Facility Study Nos. ZXAA-0007-DV-HB (Microsomes)
and ZXAA-0008-DV-IB (Hepatocytes)

STUDY SUMMARIES

The purpose of these studies was to determine the in-vitro stability of HM1010 in CD-1 mouse, Sprague-Dawley rat, beagle dog, cynomolgus monkey, and human liver microsomes and cryopreserved hepatocytes.

For ZXAA-0007-DV-HB, the test and control articles (HM1010 and verapamil) were tested at a concentration of 2 µM in pooled liver microsomes, 0.5 mg protein/mL, of each species. The test and control articles were incubated at 37°C for 120 minutes. At the assay points (time zero, 15, 30, 60, 90, and 120 minutes), samples were removed for analysis by LC-MS/MS. Calculations were performed using peak area ratios.

Microsomal stability results for HM1010 are summarized below:

Species	$T_{1/2}$ (min)	% Remaining at 120 min	CL_{int} (mL/min/kg)
CD-1 mouse	32.0	0.8	170
Sprague-Dawley rat	27.7	3.7	101
Beagle dog	33.0	1.5	47.2
Cynomolgus monkey	21.8	0.8	85.8
Human	20.3	1.4	61.5

The estimated half-life for HM1010 at 2 µM in the presence of CD-1 mouse, Sprague-Dawley rat, beagle dog, cynomolgus monkey, and human liver microsomes (0.5 mg proteins/mL) was 32.0 minutes, 27.7 minutes, 33.0 minutes, 21.8 minutes, and 20.3 minutes, respectively.

After incubation (120 minutes at 37°C) the percentage of HM1010 remaining was 0.8% in CD-1 mouse liver microsomes, 3.7% in Sprague-Dawley rat liver microsomes, 1.5% in beagle dog liver microsomes, 0.8% in cynomolgus monkey liver microsomes, and 1.4% in human liver microsomes.

The intrinsic clearance values for HM1010 were 170 mL/min/kg in CD-1 mouse microsomes, 101 mL/min/kg in Sprague-Dawley rat microsomes, 47.2 mL/min/kg in beagle dog microsomes, 85.8 mL/min/kg in cynomolgus monkey microsomes, and 61.5 mL/min/kg in human microsomes.

Results for the control (verapamil) demonstrated acceptable performance of the assay.

For ZXAA-0008-DV-IB, the test and control articles (HM1010 and verapamil) were tested at a concentration of 2 µM in liver hepatocytes, 0.5 million cells/mL, of each species. The test and control articles were incubated at 37°C for 180 minutes. At the assay points (time zero, 15, 30, 60, 120, and 180 minutes), samples were removed for analysis by LC-MS/MS. Calculations were performed using peak area ratios.

Hepatocyte metabolic stability results for HM1010 are summarized below:

Matrix	$T_{1/2}$ (minutes)	% Remaining at 30 minutes	CL_{int} (mL/min/kg)
CD-1 mouse	< 15	0.7	>1090
Sprague-Dawley rat	5.81	1.0	1260
Beagle dog	10.7	16.0	699
Cynomolgus monkey	12.1	18.5	465
Human	5.28	2.47	708

The estimated half-life for HM1010 at 2 µM in the presence of CD-1 mouse, Sprague-Dawley rat, beagle dog, cynomolgus monkey, and human hepatocytes (0.5 million cells/mL) was <15 minutes, 5.81 minutes, 10.7 minutes, 12.1 minutes, and 5.28 minutes, respectively.

After incubation (30 minutes at 37°C) in the presence of CD-1 mouse, Sprague-Dawley rat, beagle dog, cynomolgus monkey, and human hepatocytes, the percentage of HM1010 remaining was 0.7%, 1.0%, 16.0%, 18.5%, and 2.5%, respectively.

The intrinsic clearance (CL_{int}) values for HM1010 were >1090 mL/min/kg (mouse), 1260 mL/min/kg (rat), 699 mL/min/kg (dog), 465 mL/min/kg (monkey), and 708 mL/min/kg (human), respectively.

Results for the control (verapamil) demonstrated acceptable performance of the assay.

HM1010: Time-Dependent Inhibition of Cytochrome P450 Enzymes in Human Liver Microsomes

Testing Facility Study No.

ZXAA-0010-DV-TB

STUDY SUMMARIES

The purpose of this in-vitro study was to determine the time-dependent inhibitory potential of HM1010 on human cytochrome P450 (CYP) isozyme (1A2, 2B6, 2C8, 2C9, 2C19, 2D6 and 3A4) activity, using pooled human liver microsomes and a concentration-response IC_{50} method.

The study design is summarized below:

Test Article:	HM1010
Concentrations:	50.0, 33.3, 22.2, 5.55, 1.39, 0.348, 0.0870, 0.0218, 0.00545, and 0.00 (DMSO control) µM

Control Articles:	Fluvoxamine (1A2 inhibitor), Ticlopidine (2B6 inhibitor), Quercetin (2C8 inhibitor), Sulfaphenazole (2C9 inhibitor), Omeprazole (2C19 inhibitor), Paroxetine (2D6 inhibitor), and Mifepristone (3A4 inhibitor)
Concentrations:	50.0, 33.3, 22.2, 5.55, 1.39, 0.348, 0.0870, 0.0218, 0.00545, and 0.00 (DMSO control) µM
Test System:	Pooled human liver microsomes, mixed gender, at 0.1 mg/mL
Assay Buffer:	100 mM Potassium phosphate buffer, pH 7.4
CYP450 Substrates:	Phenacetin (1A2), Bupropion (2B6), Amodiaquine (2C8), Diclofenac (2C9), Mephenytoin (2C19), Dextromethorphan (2D6), Testosterone (3A4), and Midazolam (3A4)
Concentrations:	Approximate K_m concentration for each respective isozyme (within the linear range of CYP-mediated metabolism)
Cofactor:	β-Nicotinamide adenine dinucleotide phosphate (NADPH)
Replicates:	Two
Pre-incubation:	0 and 30 minutes at 37°C with and without NADPH (1 mM)
Incubation:	30 minutes at 37°C
Bioanalysis:	LC-MS/MS

No inhibition to minimal inhibition of human CYPs 1A2, 2B6, 2C8, 2C9, 2C19, 2D6, and 3A4 (midazolam and testosterone substrates) activity was observed at the highest HM1010 test concentration (50 µM) in pooled human liver microsomes (0.1 mg/mL).

With pre-incubation in the presence of 1 mM NADPH, inhibition of these CYP isozymes by HM1010 at 50 µM was less than 30%. Because full dose-response curves were not achieved, inhibition data was not curve fit to determine IC_{50} values for the assessment of time-dependent inhibition by HM1010.

The results for the positive control inhibitors are within the expected ranges, indicating acceptable performance of the assays.

HM1010: Time-Dependent Inhibition of Cytochrome P450 Enzymes in Human Liver Microsomes

Testing Facility Study No.

ZXAA-0010-DV-TB

STUDY SUMMARY

The purpose of this in-vitro study was to determine the time-dependent inhibitory potential of HM1010 on human cytochrome P450 (CYP) isozyme (1A2, 2B6, 2C8, 2C9, 2C19, 2D6 and 3A4) activity, using pooled human liver microsomes and a concentration-response IC_{50} method.

The study design is summarized below:

Test Article:	HM1010
Concentrations:	50.0, 33.3, 22.2, 5.55, 1.39, 0.348, 0.0870, 0.0218, 0.00545, and 0.00 (DMSO control) µM
Control Articles:	Fluvoxamine (1A2 inhibitor), Ticlopidine (2B6 inhibitor), Quercetin (2C8 inhibitor), Sulfaphenazole (2C9 inhibitor),

	Omeprazole (2C19 inhibitor), Paroxetine (2D6 inhibitor), and Mifepristone (3A4 inhibitor)
Concentrations:	50.0, 33.3, 22.2, 5.55, 1.39, 0.348, 0.0870, 0.0218, 0.00545, and 0.00 (DMSO control) µM
Test System:	Pooled human liver microsomes, mixed gender, at 0.1 mg/mL
Assay Buffer:	100 mM Potassium phosphate buffer, pH 7.4
CYP450 Substrates:	Phenacetin (1A2), Bupropion (2B6), Amodiaquine (2C8), Diclofenac (2C9), Mephenytoin (2C19), Dextromethorphan (2D6), Testosterone (3A4), and Midazolam (3A4)
Concentrations:	Approximate K_m concentration for each respective isozyme (within the linear range of CYP-mediated metabolism)
Cofactor:	β-Nicotinamide adenine dinucleotide phosphate (NADPH)
Replicates:	Two
Pre-incubation:	0 and 30 minutes at 37°C with and without NADPH (1 mM)
Incubation:	30 minutes at 37°C
Bioanalysis:	LC-MS/MS

No inhibition to minimal inhibition of human CYPs 1A2, 2B6, 2C8, 2C9, 2C19, 2D6, and 3A4 (midazolam and testosterone substrates) activity was observed at the highest HM1010 test concentration (50 µM) in pooled human liver microsomes (0.1 mg/mL).

With pre-incubation in the presence of 1 mM NADPH, inhibition of these CYP isozymes by HM1010 at 50 µM was less than 30%. Because full dose-response curves were not achieved, inhibition data was not curve fit to determine IC_{50} values for the assessment of time-dependent inhibition by HM1010.

The results for the positive control inhibitors are within the expected ranges, indicating acceptable performance of the assays.

HM1010: Induction Potential of Cytochrome P450 Enzymes (CYP1A2, CYP2B6 and CYP3A4) Using Human Cryopreserved Hepatocytes

Testing Facility Study No.

ZXAA-0011-DV-DB

STUDY SUMMARY

The purpose of this in-vitro study was to determine the induction potential of HM1010 on human cytochrome P450 (CYP) isoenzyme (1A2, 2B6 and 3A4) activity. The study design is summarized below:

Test Article:	HM1010
Concentrations:	2, 10, and 25 µM
Control Articles:	Omeprazole (1A2), Phenobarbital (2B6), and Rifampicin (3A4)
Concentration:	50 µM (omeprazole), 1000 µM (phenobarbital), and 20 µM (rifampicin)
Matrix:	Inducible cryopreserved human hepatocytes three single-donor lots
Assay Buffer:	InVitroGRO KHB

Substrates:	Phenacetin (1A2), Bupropion (2B6), and Testosterone (3A4)
Concentrations:	100 µM (phenacetin), 150 µM (bupropion), and 125 µM (testosterone)
Replicates:	Three
Assay conditions:	48 hours at 37°C in humidified 5% carbon dioxide incubator
Analysis:	LC-MS/MS (enzyme activity) and mRNA analysis

Induction of CYPs 1A2, 2B6 and 3A4 in human hepatocytes by HM1010 at 2, 10 and 25 µM was observed in at least one of the three single-donor lots of hepatocytes based on increases in enzyme activity or mRNA expression (CYP1A2 with 2 µM HM1010) that were >2-fold the vehicle control response.

CYP1A2 was induced by 25 µM HM1010 in three single-donor lots of hepatocytes with fold-increases in enzyme activity of 2.46, 5.98 and 3.65 and by 10 µM HM1010 in two of the three single-donor lots of hepatocytes with fold-increases in enzyme activity of 5.19 and 3.32. Based on enzyme activity, CYP1A2 was not induced by 2 µM HM1010. At this concentration, observed increases in enzyme activity relative to the vehicle control are <2-fold and are <20% of the positive control response; however, based on an increases in mRNA expression, minimal induction of CYP1A2 (2.24-fold increase) was observed in one lot of the three single donor lots of hepatocytes.

CYP2B6 was induced by HM1010 at 2, 10, and 25 µM in the three single-donor lots of hepatocytes. The induction appears concentration-dependent with mean fold-increases in enzyme activity of 3.00, 4.96, and 6.27 at 2 µM, 10 µM, and 25 µM HM1010, respectively.

CYP3A4 was induced by HM1010 at 2, 10 and 25 µM in at least one of the three single-donor lots of hepatocytes. The observed fold-increases in enzyme activity were minimal: 2.06 (one lot with 2 µM HM1010); 2.20 and 2.12 (two lots with 10 µM HM1010); and 2.07, 2.19 and 2.15 (three lots with 25 µM HM1010).

Across three single-donor lots of hepatocytes, the average stability of HM1010 over 24 hours incubation with hepatocytes for both days of incubation was 0.6% at 2 µM, 0.7% at 10 µM and 0.3% at 25 µM.

Results for the positive control articles (inducers) are within the expected ranges, indicating acceptable performance of the assays.

HM1010: Induction Potential of Cytochrome P450 Enzymes (CYP1A2, CYP2B6 and CYP3A4) Using Human Cryopreserved Hepatocytes

Testing Facility Study No.

ZXAA-0011-DV-DB

The purpose of this in-vitro study was to determine the induction potential of HM1010 on human cytochrome P450 (CYP) isoenzyme (1A2, 2B6 and 3A4) activity. The study design is summarized below:

Test Article:	HM1010
Concentrations:	2, 10, and 25 µM

Control Articles:	Omeprazole (1A2), Phenobarbital (2B6), and Rifampicin (3A4)
Concentration:	50 µM (omeprazole), 1000 µM (phenobarbital), and 20 µM (rifampicin)
Matrix:	Inducible cryopreserved human hepatocytes three single-donor lots
Assay Buffer:	InVitroGRO KHB
Substrates:	Phenacetin (1A2), Bupropion (2B6), and Testosterone (3A4)
Concentrations:	100 µM (phenacetin), 150 µM (bupropion), and 125 µM (testosterone)
Replicates:	Three
Assay conditions:	48 hours at 37°C in humidified 5% carbon dioxide incubator
Analysis:	LC-MS/MS (enzyme activity) and mRNA analysis

Induction of CYPs 1A2, 2B6 and 3A4 in human hepatocytes by HM1010 at 2, 10 and 25 µM was observed in at least one of the three single-donor lots of hepatocytes based on increases in enzyme activity or mRNA expression (CYP1A2 with 2 µM HM1010) that were >2-fold the vehicle control response.

CYP1A2 was induced by 25 µM HM1010 in three single-donor lots of hepatocytes with fold-increases in enzyme activity of 2.46, 5.98 and 3.65 and by 10 µM HM1010 in two of the three single-donor lots of hepatocytes with fold-increases in enzyme activity of 5.19 and 3.32. Based on enzyme activity, CYP1A2 was not induced by 2 µM HM1010. At this concentration, observed increases in enzyme activity relative to the vehicle control are <2-fold and are <20% of the positive control response; however, based on an increases in mRNA expression, minimal induction of CYP1A2 (2.24-fold increase) was observed in one lot of the three single donor lots of hepatocytes.

CYP2B6 was induced by HM1010 at 2, 10, and 25 µM in the three single-donor lots of hepatocytes. The induction appears concentration-dependent with mean fold-increases in enzyme activity of 3.00, 4.96, and 6.27 at 2 µM, 10 µM, and 25 µM HM1010, respectively.

CYP3A4 was induced by HM1010 at 2, 10 and 25 µM in at least one of the three single-donor lots of hepatocytes. The observed fold-increases in enzyme activity were minimal: 2.06 (one lot with 2 µM HM1010); 2.20 and 2.12 (two lots with 10 µM HM1010); and 2.07, 2.19 and 2.15 (three lots with 25 µM HM1010).

Across three single-donor lots of hepatocytes, the average stability of HM1010 over 24 hours incubation with hepatocytes for both days of incubation was 0.6% at 2 µM, 0.7% at 10 µM and 0.3% at 25 µM.

Results for the positive control articles (inducers) are within the expected ranges, indicating acceptable performance of the assays.
